# Statistical learning and adaptive decision-making underlie human response time variability in inhibitory control

**DOI:** 10.3389/fpsyg.2015.01046

**Published:** 2015-08-11

**Authors:** Ning Ma, Angela J. Yu

**Affiliations:** ^1^Department of Electrical and Computer Engineering, University of CaliforniaSan Diego, La Jolla, CA, USA; ^2^Department of Cognitive Science, University of CaliforniaSan Diego, La Jolla, CA, USA

**Keywords:** bayesian modeling, decision making, learning, response time, psychophysics, inhibitory control, stop signal task

## Abstract

Response time (RT) is an oft-reported behavioral measure in psychological and neurocognitive experiments, but the high level of observed trial-to-trial variability in this measure has often limited its usefulness. Here, we combine computational modeling and psychophysics to examine the hypothesis that fluctuations in this noisy measure reflect dynamic computations in human statistical learning and corresponding cognitive adjustments. We present data from the stop-signal task (SST), in which subjects respond to a go stimulus on each trial, unless instructed not to by a subsequent, infrequently presented stop signal. We model across-trial learning of stop signal frequency, P(stop), and stop-signal onset time, SSD (stop-signal delay), with a Bayesian hidden Markov model, and within-trial decision-making with an optimal stochastic control model. The combined model predicts that RT should increase with both expected P(stop) and SSD. The human behavioral data (*n* = 20) bear out this prediction, showing P(stop) and SSD both to be significant, independent predictors of RT, with P(stop) being a more prominent predictor in 75% of the subjects, and SSD being more prominent in the remaining 25%. The results demonstrate that humans indeed readily internalize environmental statistics and adjust their cognitive/behavioral strategy accordingly, and that subtle patterns in RT variability can serve as a valuable tool for validating models of statistical learning and decision-making. More broadly, the modeling tools presented in this work can be generalized to a large body of behavioral paradigms, in order to extract insights about cognitive and neural processing from apparently quite noisy behavioral measures. We also discuss how this behaviorally validated model can then be used to conduct model-based analysis of neural data, in order to help identify specific brain areas for representing and encoding key computational quantities in learning and decision-making.

## 1. Introduction

Response time (RT) is an oft-reported behavioral measure in psychology and neuroscience studies. As RT can vary greatly across trials of apparently identical experimental conditions, average or median RT across many identical trials is typically used to examine how task performance or an internal speed-accuracy tradeoff might be affected by different experimental conditions. Separately, a specialized subfield of quantitative psychology has used not only the first-order statistics (e.g., mean and median) but also second-order (e.g., variance) and higher-order (e.g., skewness, kurtosis) statistics to make inferences about the cognitive or neural processes underlying behavior (Laming, 1968; Luce, 1986; Smith, 1995; Ratcliff and Rouder, 1998; Gold and Shadlen, 2002; Bogacz et al., 2006). In general, RT is considered a very noisy experimental measure, with single-trial responses yielding little useful information about the underlying mental processes.

In this work, we approach RT modeling from a different angle, attempting to capture trial-to-trial variability in RT as a consequence of statistically normative learning about environmental statistics and corresponding adaptations within an internal decision-making strategy. We focus on behavior in the stop-signal task (SST) (Logan and Cowan, 1984), a classical inhibitory control task, in which subjects respond to a go stimulus on each trial unless instructed to withhold their response by an infrequent stop signal that appears some time after the go stimulus (stop-signal delay; SSD). We model trial-by-trial behavior in SST, using a Bayesian hidden Markov model to capture across-trial learning of stop signal frequency [P(stop)] and onset asynchrony (SSD), and a rational decision-making control policy for within-trial processing, which combines prior beliefs and sensory data to produce behavioral outputs under task-specific constraints/objectives.

This work builds on several previous lines of modeling research. The new model combines a *within-trial* rational decision-making model for stopping behavior (Shenoy and Yu, 2011) and an *across-trial* statistical learning model (Dynamic Belief Model; DBM) that sequentially updates beliefs about P(stop) (Yu and Cohen, 2009; Shenoy et al., 2010); it also incorporates a novel across-trial learning component, a simple version of a Kalman filter, that updates beliefs about the temporal statistics of the stop-signal onset (SSD). Using this new model, we can then predict how RT on each trial *ought* to vary as a function of the sequence of stop/go trials and SSD's previously experienced by the subject, and compare it to the subject's actual RT.

Several key elements of the combined model have previously received empirical support. For example, we showed that the rational decision-making model for stopping behavior (Shenoy and Yu, 2011), which separately penalizes stop error, go (discrimination and omission) error, and response delay, can account for both classical effects in the SST (Logan and Cowan, 1984), such as increasingly frequency of stop errors as a function of SSD and faster stop-error responses than correct go responses, as well as some recently discovered, subtle influences of contextual factors on stopping behavior, such as motivation/reward (Leotti and Wager, 2009) and the baseline frequency of stop trials (Emeric et al., 2007). We also showed that the across-trial learning model, DBM, can account for sequential adjustment effects not only in SST (Shenoy et al., 2010; Ide et al., 2013), but also more broadly in simple 2AFC (2-alternative forced choice) perceptual decision-making tasks (Yu and Cohen, 2009) and a visual search task (Yu and Huang, 2014).

The primary contribution of the current work is to extend a Bayesian model of trial-by-trial learning of P(stop) (Shenoy et al., 2010) to also account for learning about the temporal distribution SSD, and to quantify how much of RT variability can be accounted for by each of these learning components. Moreover, we expect that this extended model will be useful in identifying brain regions in encoding key computational variables in learning and decision-making.

In the following, we first describe the experimental design, then the modeling details, followed by the results; we conclude with a discussion of broader implications and future directions for research.

## 2. Materials and methods

### 2.1. Experiment

**Figure 1:**
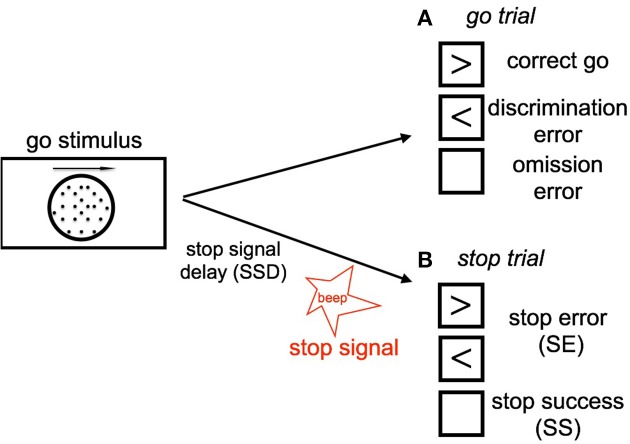
**Schematic illustration of our stop signal task (A) Go trials: On go trials, subject is supposed to make a response to a default go stimulus by pressing the left or right button, based on the coherent motion direction of random dots**. The go reaction time (Go RT) is defined as the time the subject takes to make a go response since the onset of go stimulus. The subject makes a discrimination error if he/she chooses the wrong direction (press the wrong key). Lack of response results in an omission error. **(B)** Stop trials: On small fraction of trails, a stop signal will appear and instruct the subject to withhold the go response. The time delay between the occurrence of the onset of go stimulus and stop signal is called the stop-signal delay (SSD). If the subject makes a go response in a stop trial, this trial is considered a stop error (SE) trial, otherwise it is considered a stop success trial (SS).

The stop signal task consists of a two alternative forced-choice (2AFC) perceptual discrimination task, augmented with an occasional stop signal. Figure 1 schematically illustrates our version of the SST: subject responds to a default go stimulus on each trial within 1100 ms, unless instructed to withhold the response by an infrequent auditory stop signal. The go task is either a random-dot coherent motion task (8, 15, or 85% coherence), or a more classical square vs. circle discrimination task. On a small fraction of trials, an additional *stop* signal occurs at some time (known as the stop-signal delay, or SSD) after the go stimulus onset, and the subject is instructed to withhold the *go* response. The trials without stop signals are called *go* trials. The SSD is randomly and uniformly sampled on each trial from 100, 200, 300, 400, 500, and 600 ms.

Twenty subjects (13 females) participated in the stop signal task where, on approximately 25% of trials, an auditory “stop” signal was presented some time after the go (discrimination) stimulus, indicating that the subject should withhold their response to the go stimulus. Each subject participated in 12 blocks, 3 block for each stimulus type, and each block containing 75 trials. Two days before the main experiment session, subjects participated in a training session, which contained only 2AFC discrimination and no stop trials. In the training session, there were 10 blocks, 3 blocks for each random dot stimulus coherence and one block for shape discrimination. Subjects were given the same maximal amount of time to respond on the training session trials (1100 ms) as in the main experiment. The purpose of the training session is to allow subjects to familiarize themselves with the task and to allow their perceptual discrimination performance to stabilize. Only data from the main experimental session are analyzed and presented here.

We say that the subject makes a discrimination error when he/she incorrectly responds to the stimulus in go trials, i.e., choosing the opposite motion direction or incorrect shape. The subject makes an omission error if he/she fails to make a go response prior to the response deadline on a go trial. The trials having stop signal are called stop trials; trials without stop signal are go trials. When the subject withhold the response until the response deadline on a stop trial, the trial is considered a stop success (SS) trial; otherwise, it is considered a stop error (SE) trial. Each trial is terminated when the subject makes a response, or at the response deadline itself if no response has been recorded. To incentivize the subjects to be engaged in the task, and to standardize the relative costs of the different kind of errors across individuals, subjects are compensated proportional to points they earn in the task, whereby they lose 50 points for a go discrimination or omission error, 50 points for a SE, and 3 points for each 100 ms of response delay (so maximally 33 points for a trial that terminates with no response, and less if the subject makes a response prior to the response deadline).

This study protocol was approved by the University of California San Diego Human Subjects Review Board, and all subjects gave written informed consent.

### 2.2. Model

In this section, we give a brief description of the computational model we use to capture both within-trial sensory processing and decision-making, and across-trial learning of P(stop) and SSD. The model for within-trial processing is essentially identical to that in our previous work (Shenoy et al., 2010; Shenoy and Yu, 2011), while the model for across-trial processing is an augmentation of a previous model (Ide et al., 2013) by taking into account not only P(stop) but also SSD.

#### 2.2.1. Within-trial processing

Within-trial processing is modeled as a combination of Bayesian sensory processing, which consists of iterative statistical inference about the identity of the go stimulus and the presence of the stop signal, and optimal stochastic control, which chooses whether to Wait or Go (and if so, which Go response) at each instant, based on the accumulating sensory information (Bayesian belief state) and general behavioral objectives (an objective function consisting of parameterized costs for response delay, go discrimination error, go omission error, and SE). We briefly summarize the model here; a more detailed description can be found elsewhere (Shenoy and Yu, 2011).

##### Sensory processing as Bayesian statistical inference

**Figure 2:**
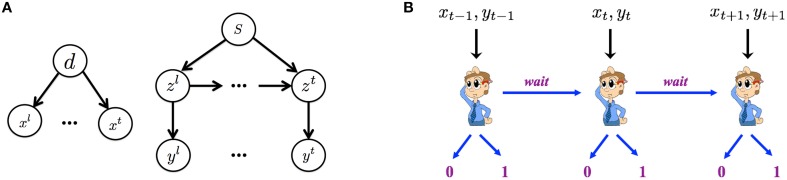
**Within-trial sensory processing and decision-making. (A)** Bayesian generative model of iid sampled sensory observations (*x*^1^, …, *x*^*t*^, …) conditioned on Go stimulus identity (*d* = 0 for left, *d* = 1 for right), and an independent stream of observations (*y*^1^, …, *y*^*t*^, …) conditioned on the presence (*z*^*t*^ = 1) or absence (*z*^*t*^ = 0) of the Stop signal, which has a geometrically distributed onset time when it is a stop trial *s* = 1 and never appears on a go trial (*s* = 0). **(B)** The decision of whether to Go, when to do so, and which Go response to select are modeled as a sequential decision-making process, where the subject chooses at each moment in time whether to select a Go response, or to wait at least one more time point.

Figure 2A graphically illustrates the Bayesian generative model for how iid noisy sensory data are assumed to be generated by the (true) hidden stimulus states. The two hidden variables *d* and *s* correspond, respectively to the identity of the go stimulus, *d* ∈ {0, 1} (0 for left, 1 for right), and whether or not this trial is a stop trial, *s* ∈ {0, 1}. Conditioned on the go stimulus identity *d*, a sequence of iid sensory inputs, representing the cue of go stimulus, are generated on each trial, *x*^1^, …, *x*^*t*^, …, where *t* indexes time steps *within a trial*. The likelihood functions of *d* generating the sensory inputs are *f*_0_(*x^t^*) = *p*(*x^t^*|*d* = 0) and *f*_1_(*x^t^*) = *p*(*x^t^*|*d* = 1), which are assumed to be Bernoulli distribution with respective rate parameters *q*_*d*_ and 1 − *q*_*d*_. The dynamic variable *z*^*t*^ denotes the presence/absence of the stop signal. *z*^1^ = … = *z*^θ − 1^ = 0 and *z*^θ^ = *z*^θ+1^ = … = 1 if a stop signal appears at time θ, where θ represents stop signal delay SSD. For simplicity, we assume that θ, also known as the stop-signal delay (SSD), follows a geometric distribution: *P*(θ = *t*|*s* = 1) = *q*(1 − *q*)^*t* − 1^. The expected value of θ is 1∕*q*, which is the expected SSD, 𝔼[*SSD*], within a trial. Conditioned on *z*^*t*^, each observation *y*^*t*^ is independently generated and indicates the cue of stop signal. For simplicity, we assume the likelihood functions, *p*(*y^t^*|*z^t^* = 0) = *g*_0_(*y^t^*) and *p*(*y^t^*|*z^t^* = 1) = *g*_1_(*y^t^*), are Bernoulli distributions with respective rate parameters *q*_*s*_ and 1 − *q*_*s*_.

In the statistically optimal recognition model, Bayes' Rule is applied in the usual iterative manner to compute the iterative posterior probability associated with go stimulus identity, *p^t^_d_* : = *P*(*d* = 1|**x**^*t*^), and the presence of the stop signal, *p^t^_s_*: = *P*(*s* = 1|**y**^*t*^), where **x**^*t*^ = {*x*^1^, *x*^2^, …, *x*^*t*^} and **y**^*t*^ = {*y*^1^, *y*^2^, …, *y*^*t*^} denotes all the data observed so far. The *belief state* at time *t* is defined to be the vector **b**^*t*^ = (*p^t^_d_, p^t^_s_*), which can be iteratively computed from time step to time step via Bayes' Rule, by inverting the generative model (Figure 2).

##### Decision making as optimal stochastic control

Figure 2B graphically illustrates the sequential decision-making process used to model how an observer chooses whether to Go, when to do so, and which Go response to select on each trial. The decision policy is optimized with respect to the Bayesian belief state and a behaviorally defined cost function that captures the cost and penalty structure of SST, based on which the observer decides at each moment in time whether to Go (and if so, which Go response) or Wait at least one more time step.

On each trial, if the Go action is taken by the response deadline *D*, it is recorded as a Go response (correct on Go trials, error on Stop trials); otherwise the trial is terminated by the response deadline and a Stop response is recorded (omission error on Go trials, correct on Stop trials). Let τ denote the trial termination time, so that τ = *D* if no response is made before the deadline *D*, and τ < *D* if a Go action is chosen. δ ∈ {0, 1} represents the possible binary Go choices produced by making a Go response. We assume there is a cost *c* incurred per unit time in response delay (corresponding to time-dependent costs, such as time, effort, opportunity, or attention), a SE penalty of *c*_*s*_ for responding on a Stop trial, and a unit cost for making a discrimination error or commission error on a Go trial—since the cost function is invariant with respect to scaling, we normalize all cost parameters relative to the Go error cost without loss of generality. Thus, the cost function is:

$$\begin{array}{l} l(\tau ,\;\delta ;\;d,\;s,\;\theta ,\;D)=c\tau +c_{s}1_{\left\{\tau <D,s\;=\;1\right\}}+1_{\left\{\tau <D,\delta \;\neq \;d,s\;=\;0\right\}}\\ \;+\;1_{\left\{\tau \;=\;D,s\;=\;0\right\}}. \end{array}$$

The optimal decision policy minimizes the expected (average) loss, *L*_π_ = 𝔼[*l*(τ, δ; *d, s*, θ, *D*)], where r refers to the baseline probability of encountering a stop trial:

$$\begin{array}{l} L_{\pi }=c�[\tau ]+c_{s}rP(\tau <D|s=1)+(1-r)P(\tau \text{ ​}<\text{ ​}D,\delta \neq d|s=0)\\ \;+\;(1-r)P(\tau =D|s=0) \end{array}$$

which is an expectation taken over hidden variables, observations, and actions, and generally computationally intractable to minimize directly. Fortunately, having formulated the problem in terms of a belief state Markov decision process, we can effectively use standard dynamic programming (Bellman, 1952), or backward induction, to compute the optimal policy and action, via a recursive relationship between the value function and the Q-factors. The value function *V*^*t*^(**b**^*t*^) denotes the expected cost of taking the optimal policy henceforth when starting out in the belief state **b**^*t*^. The Q-factors, $Q_{g}^{t}\left({\text{b}}^{t}\right)$ and $Q_{g}^{w}\left({\text{b}}^{t}\right)$, denote the minimal costs associated with taking the action Go or Wait, respectively, when starting out with the belief state **b**^*t*^, and subsequently adopting the optimal policy. The Bellman dynamic programming principle, applied to our problem, states:

$$\begin{array}{l} \;Q_{g}^{t}(b^{t})=ct+c_{s}p_{s}^{t}+(1-p_{s}^{t})\text{min}(p_{d}^{t},1-p_{d}^{t})\\ Q_{w}^{t}(b^{t})=1_{\left\{D>t\;+\;1\right\}}�{\left[V^{t\;+\;1}(b^{t\;+\;1})|b^{t}\right]}_{b^{t\;+\;1}}\\ \;+\;1_{\left\{D\;=\;t\;+\;1\right\}}(c(t+1)+1-p_{s}^{t})\\ V^{t}(b^{t})\;=\text{ min}(Q_{g}^{t},\;Q_{w}^{t}) \end{array}$$

whereby the optimal policy in state **b**^*t*^ is to choose between Go and Wait depending on which one has the smaller expected cost. Note that a Go response terminates the current trial, while a Wait response lengthens the current trial by at least one more time step (unless terminated by the externally imposed response deadline). Since the observer can no longer update the belief state nor take any action at the deadline, the value function at *t* = *D* can be computed explicitly, without recursion, as $V^{D}\left(b^{D}\right)=cD+\left(1-P_{s}^{D}\right)$. Bellman's equation then allows us compute the value functions and Q factors exactly, up to discretization of the belief state space, backwards in time from *t* = *D* − 1 to *t* = 1. In practice, we discretize the belief state space, $\left(p_{d}^{t},p_{s}^{t}\right)$, into 200 × 200 bins.

The optimal policy partitions the belief state into three discrete action regions: two symmetric *Go regions* for extreme values of *p*_*d*_ and relatively small values of *p*_*s*_ (i.e., where the subject believes the probability of a stop trial is small and the confidence about whether the go stimulus requires a left/right response is high), where the optimal action is to Go, and a large central *Wait region*, where the value of *p*_*d*_ is close to 0.5 (subject is unsure of go stimulus identity) and/or the value of *p*_*s*_ is large (subject is fairly sure of this being a stop trial), and the optimal action is to Wait.

#### 2.2.2. Across-Trial processing

Across-trial processing is modeled as Bayesian iterative inference about trial type, P(stop), and the temporal onset of the stop signal, SSD.

##### Dynamic belief model for P(stop)

**Figure 3:**
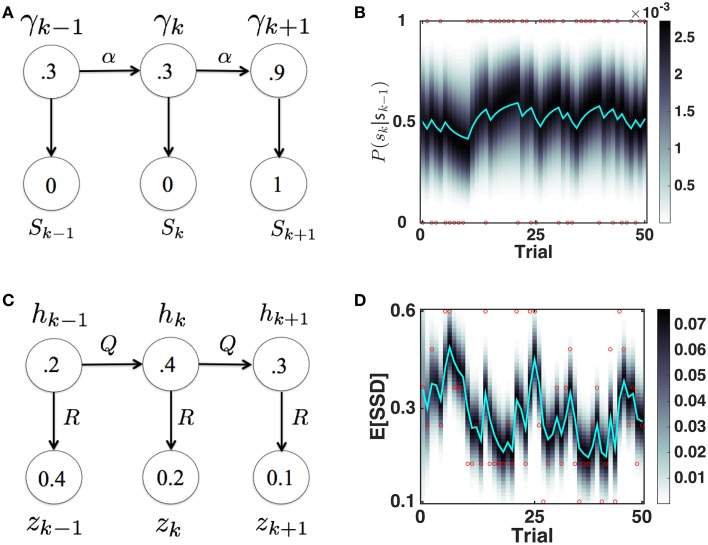
**Bayesian sequential inference model for learning *P*(*stop*) and 𝔼 [*SSD*]. (A)** Graphical model for DBM. γ ∈ [0,1], *s*_*k*_ ∈ {0, 1}. *p*(γ_*k*_|γ_*k* − 1_) = αδ(γ_*k*_ − γ_*k* − 1_)+(1 − α)*p*_0_(γ_*k*_), where *p*_0_ = *Beta*(*a, b*). Numbers inside circles indicate example random variable values. **(B)** Evolution of predictive probability mass for DBM *p*(γ_*t*_|**s**_*k* − 1_) (grayscale) and its mean, the predictive probability *P*(*s*_*k*_ = 1|**s**_*k* − 1_) (cyan), for a randomly generated sample sequence of observations (red dots valued 1 or 0). *P*(*s*_*k*_ = 1|**s**_*k* − 1_) fluctuates with transient runs of stop (e.g., starting at trial 11) and go trials (e.g., starting at trial 6). Simulation parameters: α = 0.75, *p*_0_ = *Beta*(2.5, 7.5). **(C)** Graphical model for the Kalman filter. *p*(*h*_*k*_|*h*_*k* − 1_) = 𝓝(*h*_*k* − 1_, *Q*), *p*(*z*_*k*_|*h*_*k*_) = 𝓝(*h*_*k*_, *R*), *p*(*h*_1_) = 𝓝(*h*_0_, *P*_0_). Numbers inside circles indicate example random variable values. **(D)** Evolution of posterior mean (cyan) and probability mass (grayscale) of SSD over time, for a randomly generated sequence of observations (red circles) with values in {0.1, 0.2, 0.3, 0.4, 0.5, 0.6}. 𝔼[*SSD*] tends to increase when a number of large SSD have been observed (e.g., starting at trial 6) and decrease when a number of small SSD (e.g., starting at trial 11) have been observed. Simulation parameters: *Q* = 0.03, *R* = 0.15, *h*_0_ = 0.35, *P*_0_ = 1. Unless otherwise stated, these parameters are used in all the subsequent simulation.

We originally proposed the Dynamic Belief Model (DBM) to explain sequential effects in RT and accuracy in 2AFC tasks, as a function of experienced trial history (Yu and Cohen, 2009), in particular predicting the relative probability of observing a repetition (identical stimulus as last trial) or alternation (different stimulus than last trial) on each trial. Here, as we did earlier (Ide et al., 2013), we adapt DBM to model the prior probability of observing a Stop trial (as opposed to Go trial) based on trial history (see Figure 3A for a graphical illustration of the generative model, and Figure 3B for simulated dynamics of DBM given a sequence of sample observations). We briefly describe the model here; more details can be found elsewhere (Yu and Cohen, 2009; Ide et al., 2013).

We assume that γ_*k*_ is the probability that trial *k* is a stop trial, and it has a Markovian dependence on γ_*k* − 1_, so that with probability α, γ_*k*_ = γ_*k* − 1_, and probability 1 − α, γ_*k*_ is redrawn from a generic prior distribution *p*_0_(γ_*k*_). The observation *s*_*k*_ is assumed to be drawn from a Bernoulli distribution with a rate parameter γ_*k*_. The iterative posterior and prior of γ_*k*_ can be updated via Bayes' Rule by:

$$\begin{array}{l} \;p(\gamma_{k}|s_{k})\propto p(\gamma_{k}|s_{k\;-\;1})p(s_{k}|\gamma_{k})\\ p(\gamma_{k}|s_{k\;-\;1})=\alpha p(\gamma_{k\;-\;1}=\gamma |s_{k\;-\;1})+(1-\alpha )p_{0}(\gamma_{k}=\gamma )\;. \end{array}$$

Note that the predicted value of γ_*k*_, what we call P(stop), is the mean of the predictive prior distribution: *P*(*s*_*k*_ = 1|**s**_*k* − 1_) = 𝔼[γ_*k*_|**s**_*k* − 1_] = ∫γ*p*(γ|**s**_*k* − 1_)*dγ*. Under this model, *P*(*s*_*k*_ = 1|**s**_*k* − 1_) specifies the prior probability of seeing a stop signal for within-trial sensory processing in Section 2.2.1.

##### Kalman filter model for learning expected SSD

We use a simple linear-Gaussian dynamical systems model, also known as a Kalman filter (Kalman, 1960), to model the trial-by-trial estimation of the mean and variance of the posterior and predictive prior distribution of SSD in the SST. When the prior distribution of the hidden dynamic variable is Gaussian, the dynamics is linear and corrupted by Gaussian noise, and the observations are a linear function of the hidden variable corrupted by Gaussian noise, the posterior distribution of the hidden variable after each observation, as well as the predictive prior before the next observation, are both Gaussian as well. The Kalman filter describes the statistically optimal (Bayesian) equations for updating the posterior and prior distributions.

As shown in Figure 3C, we assume that the observed SSD on (stop) trial *k, z*_*k*_, is generated from a Gaussian distribution with “true” (hidden) mean *h*_*k*_ and variance *R*, whereby *h*_*k*_ evolves from (one stop) trial to (another stop) trial under Gaussian noise, with mean 0 and variance *Q*. We also assume that the prior distribution over *h*_1_ is Gaussian, *p*(*h*_1_) = 𝓝(*h*_0_, *P*_0_). Then the predictive prior distribution $p\left(h_{k}|z_{1},\ldots ,z_{k-1}\right)=𝓝\left({ĥ}_{k}^{-},P_{k}^{-}\right)$, can be updated using iterative applications of Bayes' Rule (and consistent with Kalman filter equations) as follows:

$$\begin{array}{l} \;{\hat{h}}_{k}^{-}={\hat{h}}_{k\;-\;1}\\ P_{k}^{-}=P_{k\;-\;1}+Q \end{array}$$

and the the posterior distribution, *p*(*h*_*k*_|*z*_1_, …, *z*_*k*_) = 𝓝(*ĥ*_*k*_, *P*_*k*_) can be updated as:

$$\begin{array}{l} K_{k}=\frac{P_{k}^{-}}{P_{k}^{-}+R}\\ \;{\hat{h}}_{k}={\hat{h}}_{k}^{-}+K_{k}(z_{k}-{\hat{h}}_{k}^{-})\\ \;P_{k}=(1-K_{k})P_{k}^{-} \end{array}$$

where *K*_*k*_ is known as the Kalman gain, which depends on the relative magnitude of state uncertainty $P_{k}^{-}$ and the observation noise *R*. Note that the new posterior is a linear compromise between the predictive prior and observed data, parameterized by *K*_*k*_ (see Figure 3D for simulated dynamics of the Kalman filter given a sequence of sample observations). This constitutes a particularly simple case of the Kalman filter, as both the hidden and observed variables are scalar-valued, both the hidden dynamics (how *h*_*k*_ depends on *h*_*k* − 1_) and the emission transformation (how *z*_*k*_ depends on *h*_*k*_) are trivial, and the observer does not actively control the system. The only caveat is that on trials without a stop signal (Go trials), there is no observation for *z*_*k*_; we assume on these trials the predictive prior updates as usual and the posterior distribution is identical to the prior (i.e., the inference model is allowed to diffuse as normal, but there is no observation-based correction step). An alternative implementation is to assume that the Kalman filter is not updated on Go trials (no SSD observations). We choose to allow the Kalman filter to diffuse on Go trials, because preliminary analysis indicates that the influence of recently experienced SSD diminishes with increasing number of recent Go trials. Using this model, the prior mean ${ĥ}_{k}^{-}$ specifies the mean of the prior distribution for SSD for within-trial processing (1∕*q*) in Section 2.2.1.

## 3. Results

**Figure 4:**
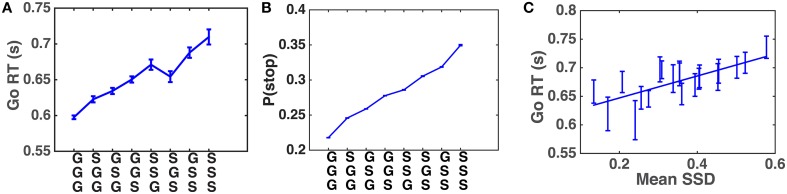
**Sequential effects in human data. (A)** Go RT increases with the frequency and recency of stop trials in recent trial history. Along the abscissa are all possible three-trial sub-sequences of Go and Stop trials: most recent trial is on the bottom. The Go RT of the correct go trial immediately following the sub-sequence is recorded. Go RT data are then averaged over all trials of a particular pattern for all subjects. Error bars indicate s.e.m. of Go RT in each pattern. **(B)** Model-predicted P(stop) increases with the frequency and recency of stop trials in recent trial history. Analogous to **(A)**, the prior P(stop) of the trial immediately following each sub-sequence is computed using DBM. Estimates of P(stop) from all trials and all subjects are then averaged in each pattern. DBM parameters: α = 0.75, *a*∕(*a*+*b*) = 0.25. **(C)** Go RT increases with experienced SSD. Go RT is plotted against mean SSD of the two most recent stop trials. A Go trial is only included if it directly follows a Stop trial (and the response was correct), and the two previous Stop trials are separated by no more than three Go trials. These restrictions are adopted because preliminary analysis indicates that the influence of recently experienced SSD diminishes with increasing number of recent Go trials. Each bin of SSD (spaced such that there are equal number of data points in each bin) contains Go RT from all trials and subjects where 𝔼[*SSD*] fell with this bin. Both Go RT and SSD are averaged within each bin. Straight line denotes best linear fit. Error bars denote s.e.m. across all trials included in data point. *R*^2^ = 0.56, *p* = 0.0002.

Systematic patterns of sequential effects have long been observed in human 2AFC tasks, in which subjects' responses speed up (and accuracy increases) when a new stimulus confirms to a recent run of repetitions or alternations, and slow down (and accuracy decreases) when these local patterns are violated (Soetens et al., 1985; Cho et al., 2002), as though humans maintain an *expectancy* of stimulus type based on experienced trial sequences and their RT is modulated by this expectancy. Similar sequential effects have also been observed in other classical behavioral experiments used in psychology, including the stop-signal task (SST), in which subjects' Go RT increases with the preponderance of stop trials in recent history (Emeric et al., 2007; Li et al., 2008). We first verify, using a relatively crude model-free method, that this effect is also present in our data. Figure 4A shows that Go RT indeed increases with the frequency of stop trials in recent history, and also with the recency of those experienced stop trials. In addition, we hypothesize that, unlike in a basic 2AFC task, subjects may maintain evolving statistical information about stimulus onset time (stop-signal delay, SSD) across trials as well. Figure 4B shows that the pattern of Go RT in Figure 4A is due to different beliefs about P(stop) resulting from different types of recent trial history, as a function of stop trial frequency and recency. Figure 4C shows that subjects' Go RT indeed increases with the mean SSD of the two most recently experienced stop trials. The strong correlation between Go RT and SSD is also consistent with recent work on decomposing decision components in the SST (White et al., 2014).

Our main modeling goal here is to develop a principled explanation for how Go RT *ought* to vary from trial-to-trial in the SST, as a function of observed data, perceived statistical structure of the environment, and behaviorally defined objectives. We can then compare model predictions with human data to see whether our assumptions about the underlying computational processes and objectives hold. There are two key components to the model (details in Section 2.2): (1) how subjects' beliefs about task statistics vary across trials as a function of previously experienced outcomes, and (2) how subjects' behavioral strategy within each trial depends on prior beliefs (learned from prior experience). These two components are generally referred to as the observation and response models (Daunizeau et al., 2010). In the context of modeling behavior, where that behavior is itself modeled under ideal Bayesian assumptions, the observer model constitutes the subject's generative model of how observations are caused, while the response model maps from the implicit beliefs to observed responses. Because we assume subjects' belief updating (Bayesian inference) and response selection are both ideal, given environmental statistics (specified by the Bayesian generative model) and behavioral objectives (specified by the objective/cost function in the stochastic control model), there are no free parameters in either the observation and response models. Furthermore, as we demonstrate through simulations, the ideal mapping between the belief state (obtained using the observation model) and the RT is essentially linear, resulting in a particularly simple parameterization of the response model.

For the first component, we separately model the evolution of subjects' beliefs about the frequency of stop trials, P(stop), using a Bayesian hidden Markov model known as the Dynamic Belief Model (DBM), and their beliefs about the temporal onset of the stop signal, SSD, using a Kalman filter model (Section 2.2.2). We previously proposed DBM to explain sequential effects in 2AFC tasks (Yu and Cohen, 2009), and later adapted it to explain sequential effects in the SST (Shenoy et al., 2010; Ide et al., 2013)—see Figure 3A for a graphical illustration of the generative model, and Figure 3B for simulated dynamics of DBM given a sequence of sample observations. To model sequential effects in SSD, we use a simple variant of the Kalman filter (Kalman, 1960). which primarily differs from DBM in that the hidden variable *s* is assumed to undergo (noisy) continuous dynamics, such that the mean of the new variable is centered at the old *s*_*k* − 1_ (a Martingale process), whereas DBM assumes that the new hidden variable *s*_*k*_ is either identical to *s*_*k* − 1_, or redrawn from a generic prior distribution *p*_0_(*s*), which is identical on each trial. This means that hidden variables dynamics in DBM are not Martingale, and the variable *s* can undergo large, discrete jumps, which are unlikely in the Kalman filter. In a preliminary analysis (results not shown), we used both the Kalman filter and a modified version of DBM (which takes continuously valued inputs instead of binary ones) to model subjects' beliefs about 𝔼[*SSD*], and found that the Kalman filter does a significantly better job of accounting for trial-by-trial variability in RT than does DBM.

For the second component, we use a Bayesian inference and optimal decision-making model (Shenoy et al., 2010; Shenoy and Yu, 2011) to predict when and whether the subject produces a Go response on each trial, as a function of prior beliefs about P(stop) and SSD. The model chooses, in each moment in time, between Go and Wait, depending on ongoing sensory data and the expected costs associated with making a go (discrimination or omission) error, a SE (not stopping on a stop trial), and response delay (details in Section 2.2.1). Our earlier work showed that this model can explain a range of behavioral and neural data in the SST (Shenoy et al., 2010; Shenoy and Yu, 2011; Ide et al., 2013; Harlé et al., 2014).

**Figure 5:**
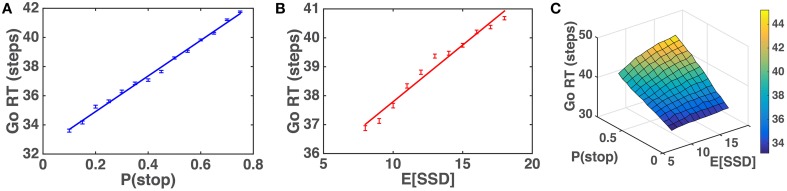
**Model prediction of Go RT vs. P(stop) and 𝔼 [*SSD*]**. **(A)** Go RT vs. P(stop): simulated Go RT for a ranged of P(stop) values (0.1, 0.15, …, 0.75). Data averaged over 10,000 simulated Go trials for each value of P(stop). Straight line denotes best linear fit. Error bars denote s.e.m. 1∕*q* = 𝔼[*SSD*] = 10. **(B)** Go RT vs. 𝔼[*SSD*]: simulated Go RT for a range of SSD values (8, 9, …, 18). Data averaged over 10,000 simulated Go trials for each value of 𝔼[*SSD*]. Straight line denotes best linear fit. Error bars denote s.e.m. P(stop) = 0.45. **(C)** Go RT vs. P(stop) and 𝔼[*SSD*]: simulated Go RT for a range of P(stop) and 𝔼[*SSD*] values, where P(stop) varies between 0.1 and 0.75, and 𝔼[*SSD*] varies between 8 and 18. Data averaged over 10,000 simulated Go trials for each [P(stop), 𝔼[*SSD*]]. Simulation parameters for A–C: *q*_*d*_ = 0.55, *q*_*s*_ = 0.72, *D* = 50, *c*_*s*_ = 0.4, *c* = 0.002. Initial string of Go trials in each block (on average 3 trials, 1/4 time none at all) are excluded from all analyses, as subjects' initial beliefs about task statistics may vary widely and unpredictably before any stop trials are observed.

We first simulate the within-trial sensory processing/decision-making model to demonstrate how the model predicts Go RT ought to vary as a function of prior beliefs about P(stop) and SSD. Intuitively, we would expect that Go RT ought to increase with the prior belief P(stop), since a higher probability of encountering a stop signal should make the subject more willing to wait for the stop signal despite the cost associated with response delay. We also expect that Go RT ought to increase with 𝔼[*SSD*] for the prior distribution, since expectation of an earlier SSD should give confidence to the observer that no stop signal is likely to come after a shorter amount of observations and thus induce the observer to respond earlier. Simulations (Figure 5) show that Go RT indeed increases monotonically with both P(stop) and 𝔼[*SSD*], and does so linearly. Note that P(stop) and 𝔼[*SSD*] are explicitly and naturally specified in the statistical model here (details in the Models section), so we only need to change these parameters and observe their normative consequences by simulating the model, without tuning any free parameters.

Given the strong linear relationship the model predicts to exist between Go RT and both P(stop) and 𝔼[*SSD*], we expect that the same would be true for human data if the across-trial learning model (Section 2.2.1) appropriately models subjects' prior beliefs about P(stop) and SSD based on experienced trial history, *and* subjects modify their internal sensory processing and decision-making accordingly as prescribed by the rational within-trial decision-making model (Section 2.2.2).

As a strong correlation between the two model predictors, P(stop) and 𝔼[*SSD*], would complicate any analysis and interpretation, we first verify that they are sufficiently decorrelated from each other (as we expect them to be, since SSD on each stop trial is chosen independently in the experimental design). We find that the average correlation between the two, across all subjects, is only 0.019 (std = 0.073), and so treat them as independent variables for the remainder of the analyses.

**Figure 6:**
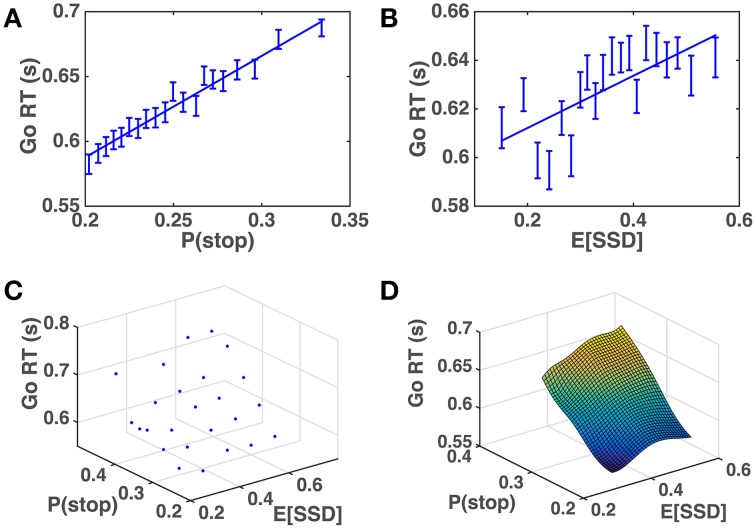
**Human Go RT vs. model-estimated P(stop) and SSD. (A)** Go RT vs. P(stop): P(stop) on each trial estimated by DBM based on actual sequence of stop/go trials the subject experienced prior to the current trial. Binning of 𝔼[*SSD*] spaced to ensure equal number of data points in each bin. Straight line denotes best linear fit of average Go RT for each bin vs. average P(stop) for each bin. Linear regression of group data: *R*^2^ = 0.97, *p* < 0.0001. Error bars denote s.e.m. DBM parameters: α = 0.75, *p*_0_ = *Beta*(2.5, 7.5). **(B)** Go RT vs. 𝔼[*SSD*]: 𝔼[*SSD*] on each trial estimated by a Kalman filter based on actual sequence of SSD the subject experienced prior to the current trial. Binning of 𝔼[*SSD*] spaced to ensure equal number of data points in each bin. Straight line denotes best linear fit between average Go RT vs. average 𝔼[*SSD*] for each bin. Linear regression of group data: *R*^2^ = 0.52, *p* = 0.0003. Error bars denote s.e.m. Kalman filter (KF) parameters: *Q* = 0.03, *R* = 0.15, *h*_0_ = 0.35, *P*_0_ = 1. **(C)** Go RT vs. P(stop) and 𝔼[*SSD*]: P(stop) and 𝔼[*SSD*] are equally discretized into 5 bins between minimum and maximum “observed” values (by applying the model to subjects' experienced sequence of trials). Each point in the grid contains RT data from all trials and all subjects where P(stop) and 𝔼[*SSD*] fell within corresponding bins. **(D)** Fitted surface plot of the scatter plot in **(C)**, by applying Matlab function *griddata*(…,′*v*4′), a biharmonic spline interpolation method, to the data in **(C)**.

We apply the across-trial learning model to a subject's experienced sequence of go/stop trials and SSD to estimate their prior beliefs on each trial, and then plot how Go RT varies with the model-based estimates of P(stop) and SSD. Figure 6 shows that the subjects' Go RT increases approximately linearly with prior P(stop) and SSD, as predicted by the model (Figure 5). For individual subjects, a linear regression of Go RT vs. binned values of P(stop) and 𝔼[*SSD*], using the same binning procedure as for the group data analysis in Figure 6C, is significant in 90% (18/20) of the subjects (*p* < 0.05), with *p* = 0.09 and *p* = 0.14 for the two remaining subjects. On average (across all subjects), we see that variability in P(stop) can explain 34.5% of the variability in the binned RT data (std = 25.0%), while the combined P(stop) and 𝔼[*SSD*] model can account for 47.2% of the variability in the binned RT data (std = 18.9%). RT variability explained by P(stop), on average, accounts for 68.3% of the variability explained by the combined model (std = 34.8%). In other words, P(stop) is a slightly more prominent predictor of RT variability, although we do see that in 25% of the subjects (5/20), 𝔼[*SSD*] was a stronger predictor of RT variability than P(stop), i.e., P(stop) accounted for less than 50% of the variance explained by the combined model.

These results imply that humans both continuously monitor and update internal representations about statistics related to stimulus frequency and temporal onset, and adjust their behavioral strategy rationally according to those evolving representations. Moreover, we can get some insight into implicit human assumptions about environmental statistics based on estimated model parameters. For DBM, we found that α = 0.75 yields the best linear fit between Go RT and P(stop) (highest *R*^2^-value), implying that subjects assume that the frequency of stop trials changes on average once every four trials (the expected duration between changes is 1∕(1 − α)). This is consistent with the α-value previously found in a DBM account of sequential effects in a 2AFC perceptual discrimination task (Yu and Cohen, 2009). We also found through simulations (results not shown) that the model fit was not very sensitive to the precise value of *a* and *b*, the parameters of the Beta prior distribution *p*_0_(γ), in that different values of (*a, b*) yield highly correlated predictions of P(stop). Thus, *a* and *b* were not optimized with respect to the data but instead fixed at *a*∕(*a*+*b*) = 0.25, equivalent to the empirical baseline frequency of stop trials, and *a*+*b* = 10. For the Kalman filter, we found that *Q* = 0.03 and *R* = 0.15 yield the best linear fit between Go RT and 𝔼[*SSD*], which implies that subjects expect on average that *h*_*k*_ will “diffuse” from trial to trial according to a Gaussian distribution with a standard deviation of $\sqrt{0.03}=0.17$ s, and that the perceived SSD is corrupted by unbiased observation noise with a standard deviation of $\sqrt{0.15}=0.39$ s. The correlation between Go RT and 𝔼[*SSD*] is not very sensitive to the other Kalman filter parameters (results not shown), *h*_0_, and *P*_0_, and thus those were specified with fixed values (see caption of Figure 3).

## 4. Discussion

In this paper, we presented a rational inference, learning, and decision-making model of inhibitory control, which can account for significant variability of human RT in the SST. Unlike most previous models that assume RT variability to be due to irreducible noise, we show that some of this variability reveals how fluctuations in experienced empirical statistics are used by human observers to continuously update their internal representation of environmental statistics and rationally adjust their behavioral strategy in response. As in many instances of (meta) Bayesian modeling of subject behavior, we find that it is sufficient to explain responses in terms of subject-specific prior beliefs. In other words, there is no single Bayes-optimal response valid across all trials, because individuals are equipped with their own priors, continually learned and dynamically evolving according to their individual experiences, and which in turn determine how each observed outcome is assimilated into posterior beliefs and how those beliefs drive observable responses. To be sure, our model is only a partial explanation of the overall RT variability. While our model is able to account for about half of the RT variability *averaged across subjects*, there is additional RT variability not accounted for by the model, which is obscured by the averaging process. Much room remains for future work to determine additional contextual and individual-specific factors that drive variabilities in RT.

In this work, we assumed each human subject is best modeled by fixed model parameters, such as the critical α parameter for tracking *P*(*stop*) and the ratio *R*∕*Q* for tracking 𝔼[*SSD*], both of which parameterize the *stability* of environmental statistics and thus determine the size of the “memory window” for using previous trials to predict the next trial. One may well ask whether human subjects in fact undergo meta-learning about these and other parameters over the time course of the experiment. The short answer is “no,” as we see no statistically discernible differences in human behavior in the first and second halves of the experiment (data not shown). This is not surprising, given that in a much simpler 2AFC task (where the cognitive demands within each trial are much lower), the first in which we successfully accounted for sequential effects as arising from tracking local statistics of the sensory environment (Yu and Cohen, 2009), we found that not only did subjects not behave differently in the first and second halves of the experiment, but that from an ideal observer point of view, meta-learning of α is much *too slow* to give rise to noticeably different behavior over the time course of one experimental session.

Separately, this work makes an important contribution to advancing the understanding of inhibitory control. Inhibitory control, the ability to dynamically modify or cancel planned actions according to ongoing sensory processing and changing task demands, is considered a fundamental component of flexible cognitive control (Barkley, 1997; Nigg, 2000). Stopping behavior is also known to be impaired in a number of psychiatric populations with presumed inhibitory deficits, such as attention-deficit hyperactivity disorder (Alderson et al., 2007), substance abuse (Nigg et al., 2006), and obsessive-compulsive disorder (Menzies et al., 2007). The work present here can help elucidate the psychological and neural underpinnings of inhibitory control, by providing a quantitatively precise model for the critical computational components, and thus informing both experimental design and data analysis in future work for the identification of neural functions. Along these lines, the current work has concrete applications in the analysis of neurophysiology data. Previously, we successfully applied the P(stop)-learning model (Shenoy et al., 2010) in a model-based analysis of fMRI data (Ide et al., 2013), and discovered that the dorsal anterior cingulate cortex (dACC) encodes a key prediction error signal related to P(stop); moreover, we found the dACC prediction-error signal is altered in young adults at risk for developing stimulant addiction (Harlé et al., 2014), a condition known to be associated with impaired inhibitory control and specifically stopping behavior. We expect that this new, extended model should be even more powerful in capturing human behavior and identifying neural correlates of the computations involved in proactive control, which is concerned with the preparation for inhibition in advance of sensory input. As we have *behaviorally validated*, trial-by-trial measures of underlying belief states, our model can be used to look for neural responses specifically correlated with these beliefs, allowing us to establish the functional neural anatomy of different sorts of probabilities and uncertainties.

Beyond specific implications for inhibitory control and response modeling, this work exemplifies an approach for leveraging apparently “noisy” experimental measures such as RT, to glean deep insights about cognitive representation and behavioral strategy in humans (and other animals). Even though our experiment did not explicitly manipulate the frequency of stop trials or the onset of the stop signal across the experimental session, subjects still used chance fluctuations in the local statistics of empirical observation to continuously modify their internal beliefs, and modulate their behavioral strategy accordingly. This raises the very real possibility that humans are constantly updating their internal model of the environment in any behavioral task, and the apparent “noise” in their behavioral output may often arise from an underlying monitoring and adaptation process, which can be brought to light by incorporating sophisticated normative modeling tools, such as the Bayesian statistical modeling and stochastic control methods used here. With the broadening use of these modeling tools, there should be exciting new possibilities for advancing the neural, psychological, and psychiatric study of learning, decision-making, and cognitive control.

## Author contributions

NM implemented all the code related to the models and data analysis, generated most of the figures, drafted the manuscript and assisted in the paper writing. AY supervised the model implementation, data analysis, and figure generation, and led the manuscript preparation.

## Funding

Funding was provided in part by an NSF CRCNS grant (BCS-1309346) to AY.

### Conflict of interest statement

The authors declare that the research was conducted in the absence of any commercial or financial relationships that could be construed as a potential conflict of interest.
